# Contemporary Data on the Status and Medical Management of Acute Heart Failure

**DOI:** 10.1007/s11886-022-01822-1

**Published:** 2022-11-16

**Authors:** Maria Anna Bazmpani, Christos A. Papanastasiou, Vasileios Kamperidis, Pantelis E. Zebekakis, Haralambos Karvounis, Andreas P. Kalogeropoulos, Theodoros D. Karamitsos

**Affiliations:** 1 First Cardiology Department, Aristotle University of Thessaloniki, AHEPA University Hospital, 1 Stilponos Kyriakides Str, 54636 Thessaloniki, Greece; 2 Division of Nephrology and Hypertension, 1St Department of Medicine, Medical School, AHEPA Hospital, Aristotle University of Thessaloniki, Thessaloniki, Greece; 3 Division of Cardiology, Department of Medicine, Stony Brook University, Stony Brook, NY USA

**Keywords:** Acute heart failure, Epidemiology, Mortality, Management

## Abstract

**Purpose of Review:**

Acute heart failure (AHF) is among the leading causes for unplanned hospital admission. Despite advancements in the management of chronic heart failure, the prognosis of AHF remains poor with high in-hospital mortality and increased rates of unfavorable post-discharge outcomes. With this review, we aim to summarize current data on AHF epidemiology, focus on the different patient profiles and classifications, and discuss management, including novel therapeutic options in this area.

**Recent Findings:**

There is significant heterogeneity among patients admitted for AHF in their baseline characteristics, heart failure (HF) aetiology and precipitating factors leading to decompensation. A novel classification scheme based on four distinct clinical scenarios has been included in the most recent ESC guidelines, in an effort to better risk stratify patients and guide treatment. Intravenous diuretics, vasodilators, and inotropes remain the cornerstone of management in the acute phase, and expansion of use of mechanical circulatory support has been noted in recent years. Meanwhile, many treatments that have proved their value in chronic heart failure demonstrate promising results in the setting of AHF and research in this field is currently ongoing.

**Summary:**

Acute heart failure remains a major health challenge with high in-hospital mortality and unfavorable post-discharge outcomes. Admission for acute HF represents a window of opportunity for patients to initiate appropriate treatment as soon as possible after stabilization. Future studies are needed to elucidate which patients will benefit the most by available therapies and define the optimal timing for treatment implementation.

## Introduction

Acute heart failure (AHF) is defined as onset or rapid deterioration of symptoms and signs of heart failure (HF) that require urgent medical attention, and most often leads to the need for unplanned hospital admission [1]. In recent years, a plethora of advancements in chronic heart failure management has occurred, improving patient outcomes. However, AHF still carries an ominous prognosis, and established therapeutic approaches have not substantially increased short and long-term survival after an episode of acute decompensation. Importantly, the early post-discharge period (i.e., first 60 to 90 days) has been characterized as a vulnerable phase with a high risk for readmission and mortality, ranging from 15 to 30% [2, 3]. Early initiation of HF treatments, shortly after stabilization and before discharge, may have beneficial effects and several studies are testing this hypothesis. In an effort to better clarify the characteristics, management and effect of treatment on outcomes of patients admitted with AHF, national, and international registries have been carried out. However, the need to improve outcomes after an episode of AHF still remains unmet. This review aims to (i) summarize current data on the epidemiology of AHF based on evidence from large registries, (ii) outline different patient profiles with AHF that impact management, and (iii) discuss current and emerging treatment options in the acute and early post-discharge period of patients with AHF.

## Contemporary Data on Epidemiology and Prognosis

Heart failure affects millions of people worldwide. It has been estimated that AHF accounts for over one million admissions annually in the United States alone, being the most frequent cause of hospitalization in the elderly [4, 5]. Large prospective registries are pivotal in guiding decision making and research since they play a crucial role in the identification of gaps and variations in clinical practice across countries. According to the latest registry reports from Europe, the United States and internationally, patients with AHF are predominantly males with a mean age ranging from 69 to 80 years [6–11] (Table 1). Comorbidities in HF populations of the western world did not vary significantly. Overall, the most commonly encountered were hypertension, coronary artery disease and diabetes, with prevalence of 65%, 50%, and 40%, respectively.

**Table 1:** Epidemiology and prognosis data from AHF registries of recent years

Registry	**ESC-HF pilot**	**EHFS II**	**EAHFE**	**ESC-EORP-HFA HF-LT**	**ALARM-HF**	**REPORT-HF**
Region	Europe	Europe	Spain	Europe	International	International
Number of patients	1892(AHF)	3580	13,971	7865(AHF)	4953	18,102
Time period	2004–2005	2004–2005	Different time points (2007/2009/ 2014/2016)	2011–ongoing	2006–2007	2014–2017
Demographics						
Age, years	69(± 13)	69.9(± 12)	80(± 10)	69 ± 12.9	66–	67(57–77)
					70(median)	
					38%	
Female	37.4%	39%	55.5%	37.1%		39%
Previous HF diagnosis	N/A	62.9%	60.4%	70.3%	63.8%	57%
Medical history						
CAD	50.7%	53.6%	29.4%	53.4%previous MI, 20.3%PCI, 10%CABG	N/A	48%
Hypertension AF DM CKD	61.8% 43.7% 35.1% 26%	62.5% 38.7% 32.8% 16.8%	83.5% 48.9% 42.2% 26.2%	N/A N/A 39% 26.3%		64% 31% 37% 20%
LVEF	64.5% (< 45%)	29.9% (< 30%)	21.6%(reduced)	51.1%(reduced)	74% (< 45%)	50% (< 40%)
	35.5% (≥ 45%)	35.8% (30–44%)	14.3%(mid-range)	25.1%(mid-range)	26% (≥ 45%)	17% (40–49%)
		34.3% (≥ 45%)	56.1%(preserved)	23.8%(preserved)		31% (≥ 50%) 9% missing
Length of stay, days	N/A	9(6–14)	9.3(± 8.6)	10.7(± 25.4)	6(4–10)	8(5–12)
In-hospital mortality	3.8%	6.7%	7.8%	5.3%	12%	N/A
Post-discharge mortality	17.2% (1-year)	N/A	10.2%(30 days) 30.3% (1-year)	22.2%(1 ear)	N/A	20%(1 ear)
Post-discharge readmissions	31.9% (1-year, all-cause) 24.8% (1-year, HF)	N/A	16.9% (30-day readmissions)	43.6% (1-year, all-cause) 25.6% (1-year, HF)	N/A	38%(all-cause) 22% (HF hospitalization) 39% (death or HF hospitalization)

*CAD* coronary artery disease, *AF* atrial fibrillation, *DM* diabetes mellitus, *CKD* chronic kidney disease, *HF* heart failure *LVEF* left ventricular ejection fraction

Despite all the advances in chronic HF management, in-hospital mortality remains high, ranging from 4 to 12%. The worst prognosis is among patients requiring admission to the cardiac care unit and administration of inotropes and/or vasopressors [12]. Post-discharge mortality rates are also high, as one out of five patients will die within a year following an admission for acute decompensation [6]. Mortality is particularly high in the early post-discharge period with 10% of patients dying in the first three months, rendering this phase extremely vulnerable. Patients with hypoperfusion at admission have the worse in-hospital prognosis as well as 1-year mortality that reaches 30% [13]. It is noteworthy that post-discharge readmission rates have remained high throughout the years with all-cause readmissions (for cardiovascular and non-cardiovascular causes) ranging from 32 to 44%. Finally, the prognosis is even more unfavorable in the case of cardiogenic shock with rates of in-hospital death up to 60% and 1-year mortality ranging from 50 to 60% with the majority of them occurring in the first two months [14, 15]. Epidemiological data of large AHF registries conducted in the western world are summarized in Table 1.

## Current Classification of Patients with AHF

Several classification schemes have been proposed to define patients presenting with AHF and aid in management and prognostication. The 2021 ESC guidelines for the diagnosis and treatment of acute and chronic HF have introduced an updated classification for AHF with four clinical scenarios and distinct algorithms for the management of each case [16].

**Fig. 1:**
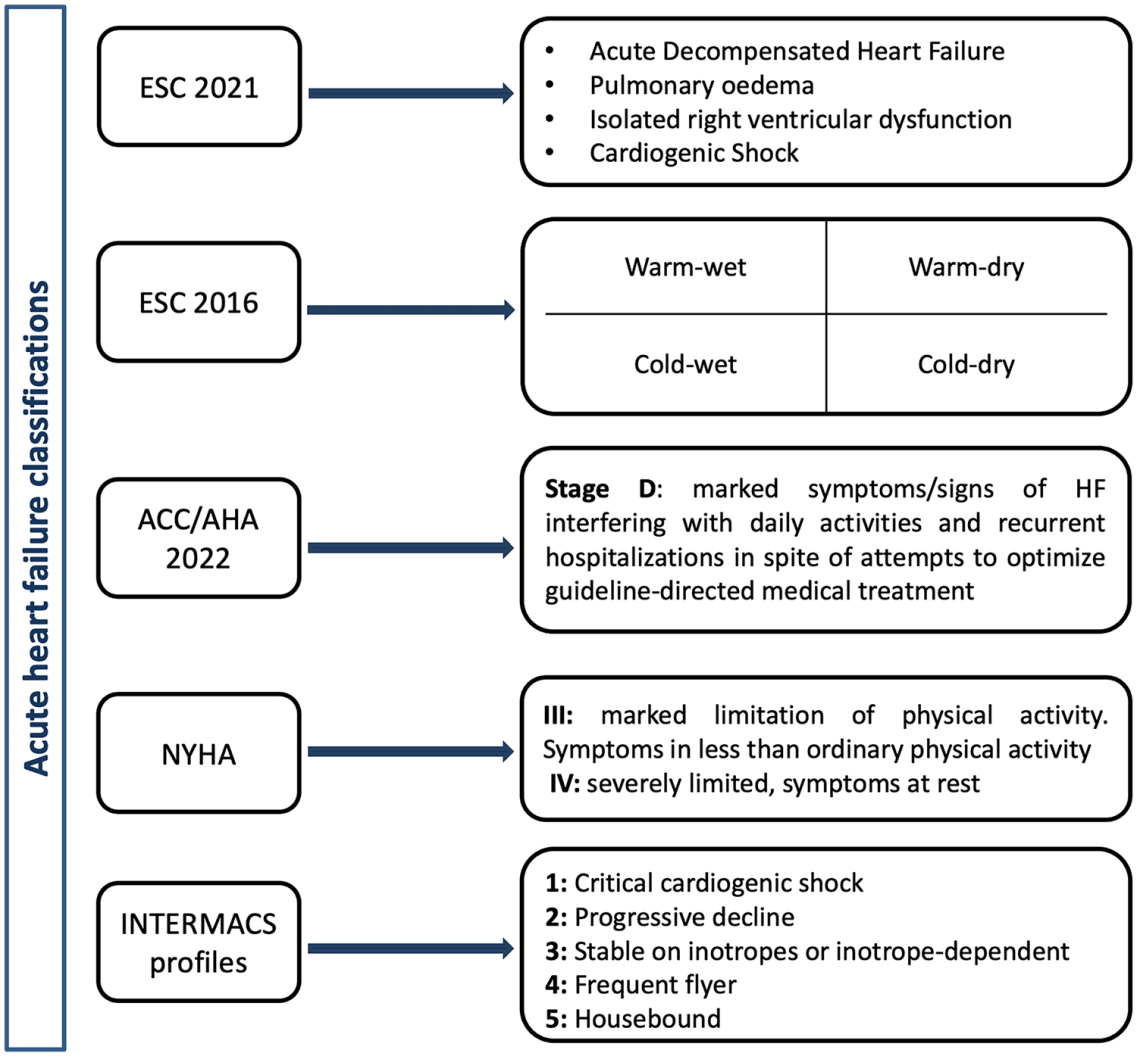
Acute heart failure classifications

The 2016 ESC clinical classification based on the presence of congestion and peripheral perfusion status has been updated by the recent guidelines, however it remains of value in patient profiling. New York Heart Association (NYHA), American College of Cardiology/American Heart Association (ACC/AHA) stages, and INTERMACS profiles are equally relevant in characterizing patients presenting with AHF. A synopsis of existing classification schemes in AHF is presented in Fig. 1.

### De Novo Vs. Decompensated HF

Based on whether a patient presents with newly diagnosed heart failure or has a pre-existing chronic heart failure history, AHF may be classified into de-novo or acutely decompensated chronic heart failure (ADHF), respectively. In both cases, the effect of precipitating factors disrupts compensatory mechanisms leading either to direct ventricular dysfunction, or to congestion development resulting in neurohormonal stimulation[17]. De novo heart failure may be further categorized into new-onset cardiac dysfunction, commonly a result of myocardial insult (infarction or inflammation causing loss of myocardial tissue), and newly diagnosed cardiac dysfunction. ADHF is more frequent than de novo with rates ranging from 50 to 75% across registries (Table 1).

### Acute Pulmonary Oedema

Acute pulmonary oedema is characterized by congestion of the lungs resulting in increased work of breathing. Large AHF registries have used different criteria for defining acute pulmonary oedema; hence, its prevalence varies significantly across registries. ESC-HF pilot and EHFS II report prevalence of 13.3 and 16.2%, respectively [6, 7]. IN-HF registry from Italy and RO-AHFS registry from Romania report prevalence of 27 and 28%, respectively [18, 19] whereas the prevalence in ALARM-HF is 37% [20]. In-hospital mortality of acute pulmonary oedema is around 5–9% in most studies [6, 9, 13, 20, 21].

### Isolated Right Ventricular Failure

The main characteristics of right ventricular dysfunction are low cardiac output leading to decreased organ perfusion and elevated central venous pressures. Right heart failure, through ventricular interdependence, impedes left ventricular filling and further reduces cardiac output[22]. The prevalence of acute right heart failure as a cause of hospital admission ranges from 2.2 to 4.5% in registries [23, 24]. Severe right ventricular dysfunction is associated with high short-term mortality and over 50% of deaths occur in the first months after diagnosis [25].

### Cardiogenic Shock

Cardiogenic shock (CS) constitutes the extreme end of AHF and is typically characterized by a state of low cardiac output, hypoperfusion of vital organs and increased lactate levels as a result of tissue hypoxia [26]. About 2–5% of AHF cases present with CS [8, 27]. In-hospital mortality is extremely high with one out of three patients with acute myocardial infarction and cardiogenic shock dying in the cardiac intensive care unit. Two clinical classifications of CS have been proposed by Chioncel and colleagues [28]: The first is a three-level approach including a pre-CS stage characterized by hypoperfusion and a systolic blood pressure (SBP) over 90 mmHg without the need for circulatory support, a CS stage characterized by a SBP of less than 90 for over 30 min or the need for pharmacologic agents or intra-aortic balloon pump in order to maintain SBP over 90 mmHg and a stage of refractory CS which is characterized by an inadequate response to treatment with two vasoactive agents. The other is an ABCDE approach taking into consideration clinical parameters, cardiac index, and lactates. In this classification, A stands for At risk of shock, B for Beginning but without hypoperfusion and with normal lactates, C for Classic shock with cardiac index < 2.2 and lactates > 2 mmol/l and the need for vasoactive agents and mechanical circulatory support, D for Deteriorating with no significant improvement after 30 min and lactates over 5 mmol/l, and finally, E stands for Extremis that results in cardiac arrest and beginning of cardiopulmonary resuscitation.

## Management

The goals of in-hospital treatment vary during the course of an AHF hospitalization. In the early phase of admission, main targets are stabilization, decongestion and/or hypoperfusion management and identification/treatment of precipitating factors. Once stabilization is warranted, minimizing length of in-hospital stay and improving prognosis should be prioritized. In hemodynamically stable patients admitted with acute heart failure, every effort to maintain guideline directed medical treatment (GDMT) should be made [29]. Upon discharge, ensuring full congestion and initiating/up-titrating GDMT are incremental for prevention of future unfavorable outcomes. At this stage, main goals are to improve overall use and adherence to GDMT. Finally, goals of long-term management include improvement in quality of life and early identification of clinical worsening.

**Fig. 2:**
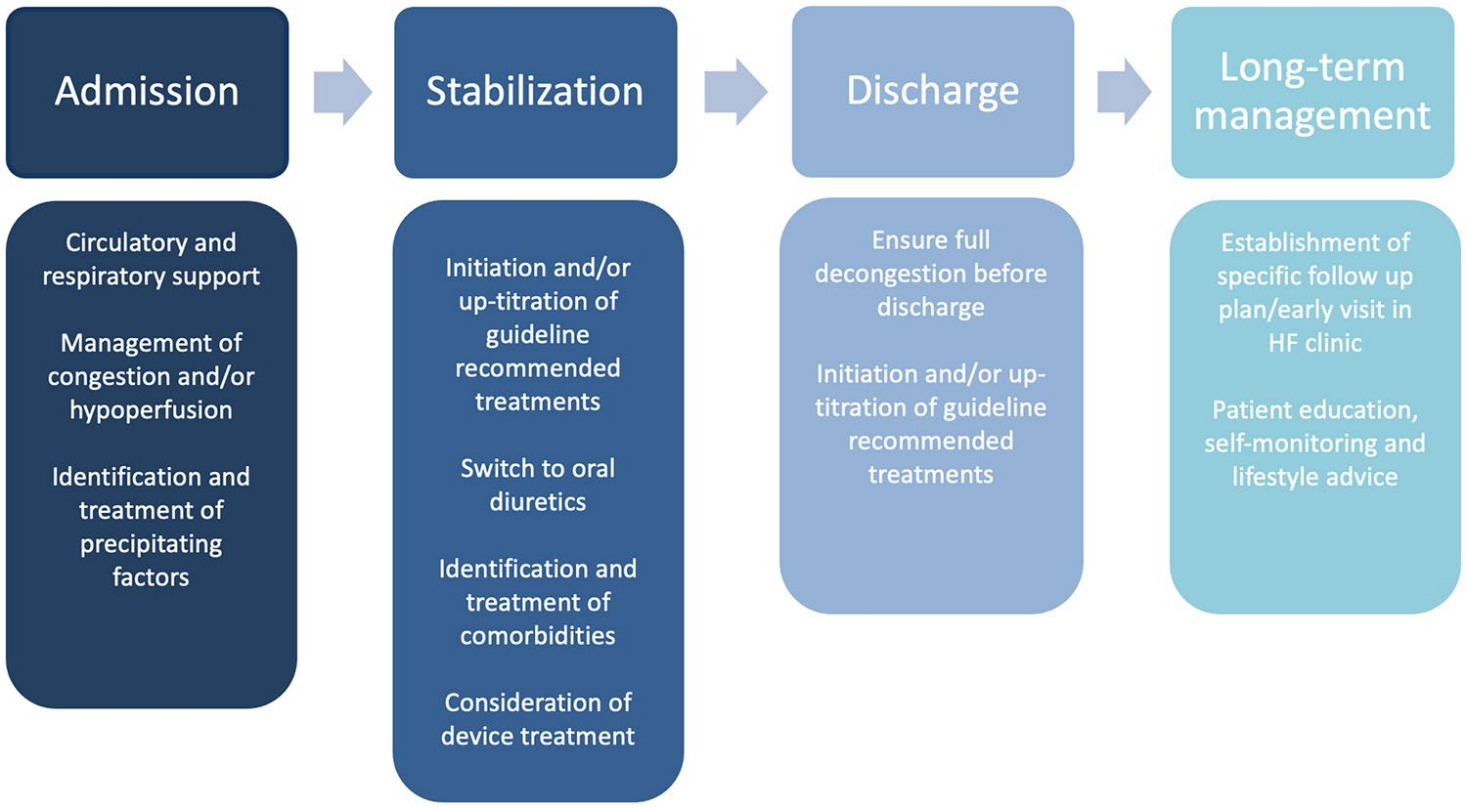
Proposed overview of management of acute heart failure patients

A proposed overview of AHF management is presented in Fig. 2.

### Admission/Initial Assessment

Clinical stabilization with preservation of end-organ function and decongestion with identification of any precipitating factors are the cornerstones of AHF treatment. At initial assessment, precipitating factors that require urgent management should be sought for and addressed. These include acute coronary syndromes, arrhythmias (tachyarrhythmias or severe bradycardia/conduction disturbances), hypertensive emergencies, acute mechanical causes and acute pulmonary embolism. Until resolution of the main precipitating factor occurs, stabilization of patient should be achieved. Early initiation of intravenous diuretics and vasoactive agents is often required at this stage and the need for mechanical circulatory support should be addressed for certain patients.

### Hemodynamic Stabilization and Mechanical Circulatory Support (MCS)

Treatment of congestion and/or hypoperfusion and maintenance of disease-modyfying therapies are among the first steps, along with oxygenation restoration, in AHF management. Diuretics and in particular loop diuretics are the foundation of AHF treatment in both the ESC and AHA/ACC guidelines and should be administered intravenously in patients with symptoms and signs of congestion [30]. In clinical practice, choice of diuretic regimen is dependent on clinician preference and the most efficient strategy remains unclear. Either continuous infusion or bolus doses of diuretics may be applied and the duration of threatment depends on patient’s status and accomplishment of euvolaemia. However, assessment of euvolemia and optimal fluid status lacks standard criteria and is mainly subjective. A proposed strategy is to evaluate initial diuretic response after 2 h with spot urinary sodium analysis and after 6 h by assessment of average urine output [31]. If deemed inadequate, increase of loop diuretics dose and/or use of combinational diuretic therapy may be applied to overcome the clinical phenomenon of diuretic resistance which is quite common in patients with AHF. A recent study, BLUSHED-AF showed that a lung-ultrasound strategy to guide treatment of pulmonary congestion led to faster decongestion in the first 48 h of hospitalization but had no benefit compared to standard care in reducing b-lines at 6 h post admission and at 30-days post-discharge [32]. For persistent volume overload, refractory to diuretic treatment, renal replacement therapy may occasionally be considered, keeping in mind that this strategy does not offer prognostic benefits [33–35]. Intravenous vasodilators may be considered as initial therapy in cases of hypertensive AHF, acute pulmonary edema and for symptomatic relief in patients with SBP > 110 mmHg [36, 37]. In cases of low output states and impaired cardiac contractility, vasoactive medications including inotropes (dobutamine, dopamine, levosimendan, and milrinone) and vasopressors (norepinephrine and epinephrine) are often required to maintain organ perfusion, bearing in mind that long-term use of these agents is potentially harmful. Hence, short-term support should be applied only for cases with severe impairment of cardiac funtion that cannot be stabilized by other measures [38–41]. In clinical practice, several issues arise when using vasoactive agents and vasopressors as on the one hand, infusion rates are often arbitrary and on the other, there is no established algorithm for weaning of these agents. Additionally, there is uncertainty in definition of clinical stability and readiness for deescalation (Table 2).

**Table 2:** Open issues in acute heart failure classification and management

**Classification**	• Several proposed classification schemes, often overlapping – may be confusing in clinical practice • Heterogenous phenotypes – troublesome interpretation of clinical trial results • Association of AHF classification and treatment options not always clear
**Management**	
Intravenous agents Diuretics Vasoactive agents/vasopressors	• Diuretic effect is variable • Uncertainty about the best dosing strategy (continuous infusion or bolus doses) • Variability in assessment of decongestion • Lack of clear recommendations on dosing and infusion rates • No clear definition of achievement of clinical stability • Lack of established algorithm for weaning
Mechanical therapies	• Use highly dependable on local availability and expertise
Pre-discharge management	• No specific criteria for readiness for hospital discharge • Optimal timing for initiation or up-titration of guideline directed medical treatment • Prognostic impact of chronic heart failure treatments in the setting of acute heart failure

In cases of AHF refractory to the measures discussed above, there is often the need for escalation of management with the use of mechanical circulatory support (MCS) as a temporary or permanent solution [42, 43]. MCS implementation varies from center to center, depending on local availability and expertise. Short-term mechanical assist devices include intraaortic ballon pump [44], Impella [45, 46], Tandem-Heart [47], and venoarterial extracorporeal membreane oxygenation (VA-ECMO) [48]. Ventricular assist devices may be used as a short-term solution, as bridge to recovery or decision for heart transplantation or as a permanent solution in highly selected patients [49]. Their indications, advantages, and limitations have been extensively described elsewhere [50•].

### Transition to Oral Treatments and Pre-Discharge Management

Once hemodynamic stabilization during AHF hospitalization is achieved, HFrEF medications should be initiated/up-titrated. Switching from intravenous to oral diuretics is often challenging. Right heart catheterization provides the most robust information concerning volume status however it is invasive and its use is not as broad as the use of non-invasive clinical and laboratory parameters such as symptoms, weight and daily urine output. Intensive diuretic treatment often results in hypovolemia which poses additional barriers in optimizing GDMT. Novel data for initiation of HFrEF treatments before discharge are presented below and most important studies in the field are summarized in Tables 3 and 4.

**Table 3:** Summary of studies on sacubitril/valsartan in AHF

	**First author**	**Study design**	**Number of patients**	**Key findings**
Sacubitril/valsartan				
	Velazquez et al. [54]	Multicenter, randomized, double blind, active controlled trial of in-hospital initiation of sacubitril/valsartan compared to enalapril	881	Time-averaged change in NT-proBNP concentration from baseline to weeks four and eight was 46.7% vs. 25.3 compared to enalapril
	Morrow et al. [55]	Exploratory analysis of PIONEER-HF assessing the clinical composite end point of HF hospitalization or CV death	881	Patients on sacubitril/valsartan had significantly lower risk for CV death or HF rehospitalization (HR 0.58; 95% CI, 0.39–0.87; *P* = 0.007) 8 weeks later
	DeVore et al. [58]	Secondary analysis of the open-label extension of PIONEER-HF	832	From week eight to twelve patients who switched from enalapril to sacubitril/valsartan had a greater decline to NT-proBNP (− 37.4%; 95% CI, − 28.1 to − 45.6; *P* < 0.001; comparing changes in 2 group)
	Velazquez et al. [59]	Secondary analysis of the PIONEER-HF	Black patients = 316 vs. white patients = 515	Black patients admitted for AHF had a similar improvement to white patients in NT-proBNP levels
	Wachter et al. [56]	Open-label randomized controlled trial	1002 pre-discharge initiation of sacubitril/valsartan = 500 post-discharge = 502	Proportion of patients achieving target dose at 10 weeks was 45.4% in the pre-discharge group and 50.7% in the post-discharge group. Early initiation of sacubitril/valsartan was safe and well-tolerated Patients were more likely to achieve target dose if they were < 65 years old, had SBP greater than 120 mmHg at baseline, de-novo HF and an estimated glomerular filtration rate > 60 ml/min/1.73 m)
	Senni et al. [60]	Subgroups analysis of the TRANSITION study	991	Achievement of target doses was higher in patients with de novo HF compared to prior HFrEF patients De novo patients had greater decrease in NT-proBNP levels at week 10
	Pascual-Figal et al. [61]	Post hoc analysis of the TRANSITION study	1002	In hospital initiation of sacubitril/valsartan led to a significantly greater reduction of NT-proBNP at discharge compared to patients who initiated post-discharge (28% vs. 4%, *P* < 0.001)
	Carballo et al. [63]	Prospective cohort study	799	In a real-world cohort of AHF patients only 15% were eligible for sacubitril/valsartan based on criteria from large RCTs
	Carcinelli et al. [64]	Retrospective observational cohort study	4666	Among patients eligible for sac/val after an episode of ADHF, only 9.2% were actually discharged on sac/val while the rest were less likely to initiate sac/val in the year following the index hospitalization
	Carcinelli et al. [65••]	Retrospective observational cohort study	897	Patients discharged on sacubitril/valsartan had high adherence 3 months after discharge Patients with high adherence had 47% lower risk for all-cause mortality and 31% lower risk for all-cause rehospitalization

**Table 4:** Selection of studies of novel medical therapies for acute decompensated heart failure

	**First author**	**Number of patients**	**Key findings**
B-blockers			
	Gattis et al. [52]	363	At 60-day post-discharge, 91.2% of patients randomized to predischarge carvedilol were on b-blocker compared to 73.4% randomized to post-discharge initiation(*P* < 0.0001). Serious adverse events did not differ between the two groups
	Fonarrow et al. [62]	2,373	At 60- to 90-day post-discharge, continuation of b-blocker was associated with lower risk for mortality or readmission (OR: 0.69; 95% CI: 0.52–0.92, *P* = 0.012) compared with no b-blocker
SGLT2inhibitors			
	Damman et al. [71]	81	In patients with AHF, initiation of empagliflozin no significant difference in dyspnoea, diuretic response, change in natriuretic peptides and length of stay was noted Reduced combined endpoint of in-hospital worsening HF, rehospitalizations for HF and death at 60 days was observed compared to placebo [4 (10%) vs. 13 (33%); *P* = 0.014] Empagliflozin increased urine output until day 4 of hospitalization [difference 3449 (95% CI 578–6321) mL; *P* < 0.01]
	Cox et al. [73•]	347	Patients with T2DM hospitalized for AHF who continued empagliflozin as part of their in-hospital antihyperglycemic regimen had better glycemic control with less hypoglycemic episodes, increased urine output and lower NT-proBNP levels Adverse events, length of stay, and in-hospital mortality did not differ between the two groups
	NCT04363697 [74]	86	Patients who continued SGLT2 inhibitors during hospitalization had fewer re-hospitalizations compared to the discontinued group (24% versus 39%, *P* = 0.008) with a hazard ratio of 0.29 (95% confidence interval 0.10–0.85)
	ClinicalTrials.gov [75]	158	In very old patients with T2DM hospitalized with AHF continuing empagliflozin reduced NT-proBNP (1699 ± 522 vs. 2303 ± 598 pg/ml, *P* = 0.021) and increased urine output
	Ferreira et al. [76]	102	Canagliflozin initiation before discharge in T2DM patients with AHF reduced NT-proBNP and readmission rated for HF (22.2% vs. 37.3%, HR: 0.45; 95% CI: 0.21–0.96; *P* < 0.039)
MRAs			
	Tost et al. [81]	360	High-dose spironolactone in patients with AHF did not significantly reduce NT-proBNP compared to usual care. No significant difference on 30-day all-cause mortality or HF hospitalizations was observed
	Jankowska et al. [83]	3717	Percentage of HF readmissions was significantly lower in the MRA group than in the no MRA group (18.7% vs. 24.8%; HR, 0.70; 95% CI, 0.60–0.86; *P* < .001) No significant difference in mortality was found between the 2 groups (15.6% vs. 15.8%; HR, 0.98; 95% CI, 0.82–1.18; *P* = 0.85)
Ferric carboxymaltose			
	McMurray and Packer [86]	1132	Treatment with ferric carboxymaltose in patients with iron deficiency stabilized after an episode of AHF reduced the risk for HF hospitalizations (RR 0.8, 95% CI 0.64–1, 0 = 0.05)

*AHF* acute heart failure, *SGLT2i* sodium glucose transporter 2 inhibitors, *HF* heart failure, *MRA* mineralocorticoid receptor antagonists, *NT-proBNP* pro B-type natriuretic peptide, *T2DM* type II diabetes mellitus

#### b-Blockers

Dose reduction or withdrawal of b-blockers is a common practice in evidence of shock, severe pulmonary edema with hemodynamic instability and requirement for inotropes and vasopressors. On the other hand, in hemodynamically stable patients with acute decompensation of chronic heart failure, maintaining b-blockers appears to be safe, well-tolerated and with prognostic benefits. This strategy has been investigated in a systematic review and meta-analysis which demonstrated a clear reduction in risk of in-hospital and short-term mortality as well as short-term rehospitalizations for patients who continued b-blockers whilst hospitalized [51]. A randomized clinical trial, IMPACT-HF, showed that pre-discharge initiation of carvedilol led to a significantly higher percentage of treatment attainment at 60-day post-randomization [52] and data from the large observational OPTIMIZE-HF registry showed a 31% lower risk for mortality and rehospitalization at 60–90 days for patients who maintained b-blockers during hospitalization [53]. Based on existing evidence, b-blockers should not be routinely withdrawn in case of AHF admission and attempts to initiate and/or up-titrate b-blocker treatment should be made once patient is hemodynamically stable.

#### Sacubitril/Valsartan

Two relatively recent studies have evaluated the use of sacubitril/valsartan in patients admitted with AHF. PIONEER-HF demonstrated that the reduction of NT-proBNP with in-hospital initiation of sacubitril/valsartan was significantly greater in comparison to enalapril and this beneficial effect was prominent from week 1 [54]. For patients to be eligible for the study, haemodynamic stability was a prerequisite and was defined by the following criteria: (i) systolic blood pressure (SBP) greater than 110 mmHg for 6 h prior to randomization, (ii) stable dose of iv diuretics and no use of IV vasodilators for the last 6 h and (iii) no use of iv inotropes in the last 24 h. In PIONEER-HF, the rates of worsening renal function, hyperkalemia, and symptomatic hypotension did not differ between the two groups. A following exploratory analysis of clinical outcomes in the PIONEER-HF study, sacubitril/valsartan reduced the risk for the composite endpoint of all-cause death and HF readmissions by 42% compared to enalapril and led to a 36% risk reduction for HF rehospitalizations [55].

The TRANSITION study also investigated in-hospital or early post-discharge initiation of sacubitril/valsartan in stabilized patients admitted with ADHF [56]. Early initiation was well tolerated in both groups and maintenance of target dose 10-week post-randomization was comparable between the two groups. In light of these two clinical trials, an expert consensus position paper provided a comprehensive algorithm for in-hospital initiation of sac/val as well as for management of hypotension [57]. Ntalianis et al. proposed four criteria for determination of clinical stability: (i) SBP equal to or greater than 100 mmHg for 6–12 h prior to the initiation, (ii) euvolemia, (iii) stable dose of intravenous diuretics for the past 6–12 h or preferably oral diuretics, (iv) no need for intravenous vasodilators, vasopressors, or inotropes for the last 6–12 h. In patients with SBP ≥ 100 mmHg, the lower dose of 24/26 mg should be preferred for initiation. Despite the encouraging findings of PIONEER-HF and TRANSITION, data derived from real-world show that in unselected HF patients, there are some limitations concerning in-hospital admission of sacubitril/valsartan (Table 3) [63, 64]. However, patients who are discharged on sacubitril/valsartan attain increased adherence and lower risk for all-cause mortality and rehospitalizations for HF [65••].

Several clinical trials assessing sacubitril/valsartan initiation in patients hospitalized for acute exacerbation of HF are on-going. PREMIER is an investigator-initiated, randomized controlled study aiming to evaluate the effect of sac/val initiation in patients with AHF versus conventional treatment on NT-proBNP concentrations in 8 weeks post-discharge [66]. PARAGLIDE-HF is a multicentre, randomized, double-blind study to assess safety and tolerability of sac/val versus valsartan as well as its effect of NT-proBNP and outcomes in patients with HFpEF who have been stabilized during hospitalization and initiated sac/val in-hospital or within 30-day post-discharge [67].

Based on existing evidence, in-hospital initiation of sacubitril/valsartan appears to be safe and is well-tolerated by most patients. Hospitalization for AHF is a window of opportunity for switching from angiotensin converting enzyme inhibitors to sacubitril/valsartan as this strategy improves overall patient adherence and eventually short and long-term prognosis.

Most important studies on sacubitril/valsartan in the setting of AHF are presented in Table 3.

#### Sodium-Glucose Co-Transporter 2 (SGLT2) Inhibitors

The beneficial effects of SGLT-2 inhibitors on clinical outcomes in patients with chronic heart failure and reduced ejection fraction have been firmly established [68, 69]. In 2021, ESC guidelines for heart failure, empagliflozin, or dapagliflozin are recommended for all patients with HFrEF, irrespectively of the presence of type 2 diabetes mellitus (T2DM), to reduce the risk of death and HF hospitalizations. Furthermore, in the EMPEROR-preserved trial, empagliflozin reduced the composite endpoint of HF hospitalizations and death, making empagliflozin the first evidence-based treatment in HFpEF [70].

Recently, the EMPA-RESPONSE-AHF study, a randomized, double-blind, placebo controlled, multicentre pilot study investigated the safety and efficacy of empagliflozin in patients with AHF [71]. According to their findings, while there was not a significant difference in the primary endpoint (change in dyspnea, diuretic response, change in natriuretic peptides and length of stay), initiation of empagliflozin was safe, in terms of renal function and systolic blood pressure and led to a reduction of in-hospital worsening, death and hospital readmission within 60 days [72]. A few recent real-world prospective studies support that continuation of SGLT2 inhibitors in diabetic patients admitted for AHF comprises a safe strategy, even in very old patients, results in greater NT-proBNP reduction and leads to fewer adverse events although larger randomized controlled studies are warranted to verify this hypothesis [73•, 74–76].

Several larger RCTs evaluating safety and efficacy of SGLT2 inhibitors in acute heart failure are currently ongoing: DICTATE-AHF [73•], DAPA ACT HF-TIMI 68 [74], Dapagliflozin Heart Failure Readmissions [74], and EMPULSE [75]. Studies on SGLT2 inhibitors in AHF are presented in Table 4.

#### Mineralocorticoid Receptor Antagonists

The beneficial effects of mineralocorticoid receptor antagonists (MRAs) in heart failure with reduced ejection fraction are unequivocal. However, in the setting of AHF there is less robust evidence deriving from a few, relatively small, studies. Initially, a pilot study by Carvalho et al. demonstrated that spironolactone administration in AHF was safe and led to faster decongestion as well as significant reduction of NT-proBNP levels [76]. A following randomized, double blind, clinical trial, the ATHENA-HF, did not verify the above findings. While treatment with high-dose spironolactone in patients with AHF was found to be safe and well-tolerated, no significant benefits in NT-proBNP levels, congestion, urine output, weight loss, or clinical outcomes were observed compared to standard of care treatment [77]. In a post hoc analysis of the ALARM-HF population, administration of MRA in patients admitted with AHF was associated with better in-hospital outcomes. However, this was an observational study and the exact doses of MRAs were not recorded [78]. In a large study from Japan which investigated the association of MRA prescription before discharge and clinical outcomes (all-cause mortality and hospital readmissions), no difference in all-cause mortality was observed [79]. The efficacy and safety of early initiation of eplerenone in patients with AHF were evaluated by a multicentre, double-blind RCT, the EARLIER trial. The study demonstrated that early eplerenone initiation was safe; however, no difference in the incidence of cardiovascular death or first readmission for HF was observed [80]. Finally, a sub-analysis of the EAHFE study evaluating outcomes of patients with HFpEF discharged on neurohormonal antagonists after an ADHF hospitalization showed that MRAs alone or in combination with other antineurohormonal drugs did not result in reduction of 1-year all-cause mortality or all-cause death and HF readmissions at 90-day post-discharge [81]. In conclusion, based on existing evidence, there is no clear advantage other that a guideline directed therapy is initiated and this likely improves adherence to treatment.

#### Intravenous Iron Supplementation

AFFIRM-HF, a large, randomized, double blind RCT, demonstrated that in patients with EF < 50% and iron deficiency stabilized after an episode of AHF, ferric carboxymaltose was safe and effective and reduced the risk of HF readmissions, however it did not affect the risk of cardiovascular death [82]. A recent substudy of AFFIRM-HF additionally demonstrated improvement in quality of life lasting up to 24-month post-administration [83]. Based on these findings, ferric carboxymaltose received a IIa recommendation for pre-discharge administration by the 2021 ESC guidelines on HF. Recently, a retrospective study evaluated intravenous sodium ferric gluconate complex administration in patients with ADHF, including patients with HFpEF. The study did not show a benefit in readmission rates compared to patients who did not receive iron supplementation [84]. Three more clinical trials, HEART-FID (NCT03037931), FAIR-HF2 (NCT03036462), and IRON-MAN (NCT02642562), are expected to shed light on the role of ferric carboxymaltose in heart failure.

### Strategies of Initiation of GDMT

The use of four foundational heart failure treatments including ACEi/ARNIs, b-blockers, MRAs, and SGLT2 inhibitors is supported by recent guidelines. Several strategies have been proposed for initiation and/or up-titration of HFrEF therapies with the most recent ones advocating rapid initiation of multiple therapies as early as possible [85, 86]. Up to recently, sequencing of HFrEF therapies has been the commonest strategy for initiation of GDMT. Traditionally, ACEi was introduced first, followed by b-blocker and MRA and if a patient remained symptomatic, switching to ARNI was attempted. However, this strategy has been demonstrated to pose significant delays in optimization of heart failure therapies. Up-titration of a drug category before moving to the addition of another GDMT may require a minimum of six months, which is not acceptable, taking into consideration that clinical benefit is observed within 1 month of treatments initiation for each of the four fundamental HFrEF treatments [87, 88]. A recent multinational observational study demonstrated that the time after a recent heart failure hospitalization to initiate a GDMT was longer for newer GDMT (ARNIs and SGLT2 inhibitors), highlighting that sequential strategy may often lead to clinical inertia [89]. Novel initiation/up-titration strategies have been proposed to overcome these problems. According to Greene et al., a simultaneous initiation of low doses of all the four foundational HFrEF treatments can be endeavored, followed by up-titration based on patient tolerability [85]. A potential pitfall of this strategy is that in case of a side effect, it would be difficult to determine which therapy caused it. McMurray and Pucker suggest an alternative 3-step approach with initiation of b-blocker and SGLT2 at the same time, followed by ARNI and MRA in a time frame of 4 weeks. After all four categories have been introduced, up-titration to target doses should be attempted thereafter [86]. Miller et al. proposed a cluster base scheme by which patients categorized as having volume overload should initiate SGLT2i and diuretics first, patients with elevated blood pressure should be started on ARNIs plus MRA and patients with elevated heart rate should initiate b-blocker along with ivabradine. In all cases, initiation of all four therapies in low doses should be achieved within 3 to 6 weeks [90]. While all these algorithms are helpful in guiding HFrEF therapy, in the real-world clinical care, initiation and up-titration of treatments are greatly individualized based on whether a patient is stable, treatment naïve or hospitalized for acute decompensation and considering patient comorbidities. In the setting of acute heart failure, for example, initial interruption of ACEi may be required upon admission while, generally, b-blockers should be maintained throughout the hospital stay. Once hemodynamic stabilization has occurred, ARNI and SGLT2 inhibitors should be promptly initiated followed by MRAs before discharge [91••], ensuring that all patients without contraindications will leave hospital with all four lifesaving treatments.

### Long-Term Management

Patient education and establishment of a specific follow up plan are the mainstays of long-term management of AHF. Detailed information on self-assessment of symptoms, monitoring of blood pressure and body weight and dietary restrictions should be provided prior to discharge. An early visit to a HF clinic should be scheduled for up titration of GDMT and consideration of device therapy and other advanced therapies that may be required in the trajectory of HF. Telemonitoring has been applied for HF patients with some encouraging results on all-cause mortality and HF hospitalizations as shown by a meta-analysis of systematic reviews on the topic, however large RCTs are still lacking in this area [92]. A few new technologies such as implantable wireless hemodynamic monitors and implantable intrathoracic impedance monitoring timely detect imminent decompensation allowing for adjustments to medical treatment thus preventing clinically significant deterioration [93–95]. Some devices are already implemented in clinical practice while others are still under investigation and their use is expected to broaden in the future.

## Conclusions, Unmet Needs, and Gaps in Knowledge

Acute heart failure remains a major challenge with high in-hospital mortality and poor post-discharge outcomes. Up to date, randomized clinical trials for medications administered in the initial phases of AHF have yielded neutral results and several issues in terms of AHF management remain to be addressed (Table 2). The selection of the best timing to administer a drug under investigation is unclear and the different phenotypes of patients presenting with AHF renders the interpretation of trial results troublesome. A precise definition of clinical stabilization is lacking, resulting in uncertainties as to what is the optimal time to initiate or uptitrate guideline directed medical treatment. Furthermore, implementation of mechanical therapies is variable, depending on local availability and expertise. In this context, optimization of disease-modifying therapies has proved to be the most significant tool in preventing readmissions and deaths. Taking this into consideration, admission for AHF represents a window of opportunity for patients to initiate GDMT as soon as possible after stabilization and to be referred to specialist HF-clinics thereafter for further optimization. Future prospective studies are warranted to elucidate which patients will benefit the most by available therapies and define the optimal timing for treatment implementation, in an effort to improve clinical outcomes in AHF.
